# Genetic dissection of grain iron and zinc, and thousand kernel weight in wheat (*Triticum aestivum* L.) using genome-wide association study

**DOI:** 10.1038/s41598-022-15992-z

**Published:** 2022-07-20

**Authors:** Gopalareddy Krishnappa, Hanif Khan, Hari Krishna, Satish Kumar, Chandra Nath Mishra, Om Parkash, Narayana Bhat Devate, Thirunavukkarasu Nepolean, Nagenahalli Dharmegowda Rathan, Harohalli Masthigowda Mamrutha, Puja Srivastava, Suma Biradar, Govindareddy Uday, Monu Kumar, Gyanendra Singh, Gyanendra Pratap Singh

**Affiliations:** 1 ICAR-Indian Institute of Wheat and Barley Research, Karnal, India; 2 ICAR-Sugarcane Breeding Institute, Coimbatore, India; 3 ICAR-Indian Agricultural Research Institute, New Delhi, India; 4 ICAR-Indian Institute of Millets Research, Hyderabad, India; 5 Punjab Agricultural University (PAU), Ludhiana, India; 6 University of Agricultural Sciences, Dharwad, India; 7 ICAR-Indian Agricultural Research Institute, Gauria Karma, Jharkhand India

**Keywords:** Plant sciences, Plant breeding

## Abstract

Genetic biofortification is recognized as a cost-effective and sustainable strategy to reduce micronutrient malnutrition. Genomic regions governing grain iron concentration (GFeC), grain zinc concentration (GZnC), and thousand kernel weight (TKW) were investigated in a set of 280 diverse bread wheat genotypes. The genome-wide association (GWAS) panel was genotyped using 35 K Axiom Array and phenotyped in five environments. The GWAS analysis showed a total of 17 Bonferroni-corrected marker-trait associations (MTAs) in nine chromosomes representing all the three wheat subgenomes. The TKW showed the highest MTAs (7), followed by GZnC (5) and GFeC (5). Furthermore, 14 MTAs were identified with more than 10% phenotypic variation. One stable MTA i.e. *AX-95025823* was identified for TKW in both E4 and E5 environments along with pooled data, which is located at 68.9 Mb on 6A chromosome. In silico analysis revealed that the SNPs were located on important putative candidate genes such as *Multi antimicrobial extrusion protein, F-box domain, Late embryogenesis abundant protein, LEA-18, Leucine-rich repeat domain superfamily,* and *C3H4 type zinc finger protein,* involved in iron translocation, iron and zinc homeostasis, and grain size modifications. The identified novel MTAs will be validated to estimate their effects in different genetic backgrounds for subsequent use in marker-assisted selection. The identified SNPs will be valuable in the rapid development of biofortified wheat varieties to ameliorate the malnutrition problems.

## Introduction

Over three billion global population suffers from diseases associated with micronutrient deficiencies including iron and zinc and the problem is more severe in the countries where the food habits are dominated by cereal-based diets^1^. Iron, zinc, and vitamin A are the three nutrients that are recognized as limiting factors in the diet by the world health organization^2^. Approximately, one-fourth of the population around the globe is suffering from anemia due to iron deficiency^3^. The women of reproductive age are more vulnerable as one in every three women is anemic, which lead to 0.12 million deaths and a loss of 48.2 million disability-adjusted life years (DALY) in 2010^4^. Anemia due to acute iron deficiency, particularly in children, pregnant, and lactating women lead to life-threatening health complexes such as chronic heart disease, kidney failure, and inflammatory bowel disease^5^. Zinc is another important micronutrient essential for various immunological and biochemical functions and severe deficiency may lead to impaired growth and development, altered immunity, pregnancy issues, and neuro-behavioral difficulties^6^. Around 17% of the world’s population is suffering from diseases related to zinc deficiency^7^, which leads to 97,330 deaths and a loss of 9.1 million DALY’s in 2010^4^. Micronutrient deficiency is the major risk factor for health loss in developing countries and the most vulnerable groups are pregnant women and children^8^.

Wheat is one of the most widely cultivated cereals and plays a key role in global food and nutritional security. Although wheat is nutritionally rich as compared to the other two major cereals (rice and maize), still most wheat-based diets fail to meet the required quantity of essential nutrients including iron and zinc. The problem of micronutrient malnutrition can be overcome by food fortification, supplementation, and diet diversification, but were unsustainable in a long run. The affordability and accessibility, particularly for the rural poor in remote areas are the other shortcomings associated with the above-mentioned approaches^9^. Therefore, enhancement of the nutritional value of crops through conventional and molecular approaches, termed as “biofortification”, has been recognized as an economical and sustainable strategy to reduce the problems associated with micronutrient and protein malnutrition.

Genetic dissection of complex quantitative traits through trait mapping approaches is essential for developing better marker-assisted breeding and genomic selection strategies. The identification of linked molecular markers governing complex traits is highly useful and economical for trait improvement, especially in the post-genomics era where the genotyping costs become much cheaper. The quantitative inheritance of wheat quality traits and significant effects of environment and genotype-environment interaction (GEI) on the expression of GFeC GZnC and TKW were documented in several studies^10–13^. In the past decade, extensive efforts have been made to identify QTLs associated with GFeC and GZnC^14–28^, and TKW^25,26,29–33^ in wheat through bi-parental populations based QTL mapping. However, QTLs identified in such approaches had a low resolution due to the restricted number of crossovers. In contrast, the mapping resolution could be greatly increased by using linkage disequilibrium (LD)-based association mapping approach where the mapping population represents a more diverse gene pool and considers historical recombination events^34^. This approach allows the detection of non-random associations of genome-wide markers with the phenotype^35^ and has been used widely to detect the markers associated with the genomic regions governing complex traits in crop plants^36^. The QTL resolution in association mapping has been significantly improved by using unrelated diverse genotypes that have accumulated many historical crossover events since their last common progenitors diverged^37^.

Although many GWAS studies have been performed for various agro-morphological traits, only a limited number of studies were conducted for nutritional quality traits in wheat. Furthermore, hexaploid wheat has a genome size of ~ 17 Gb^38^, and LD decay has not been well characterized. Alomari et al*.*^39^ identified 40 MTAs for GZnC covering all the three wheat subgenomes in a panel of 369 genotypes using a high-density SNP array. Similarly, Bhatta et al*.*^40^ used a diversity panel of synthetic hexaploid wheat (SHW), being a great reservoir of grain micronutrients, to identify 92 MTAs for 10 micronutrients including GFeC and GZnC. Velu et al*.*^41^ reported 39 MTAs for GZnC in a set of 330 bread wheat genotypes phenotyped in a wide range of environments. Liu et al*.*^42^ identified 14 significant MTAs for GFeC and GZnC, and manganese in a panel of 161 wild emmer-derived advanced lines. Genetic dissection of micronutrients including GFeC and GZnC has been performed in a diverse HarvestPlus association mapping panel consisting of 330 genotypes from CIMMYT’s biofortification breeding program^43^. A total of 16 loci were identified which are associated with the GZnC on 11 different chromosomes covering all three wheat subgenomes in a set of 246 wheat varieties^44^. Similarly, Calderini et al*.*^45^ used a set of 167 *Ae. tauschii* accessions to map nine MTAs governing GFeC and GZnC^46^. A total of 29 unique loci associated with grain GZnC was identified in a diversity panel of 207 bread wheat genotypes^47^.

The TKW has no nutritional value per se in wheat, however, it has a dilution effect on protein and micronutrients. Therefore, TKW is one of the important breeding objectives due to its twin effects on yield and protein. The MTAs have been identified for TKW^48–51,91^ using different compositions of GWAS panels. Therefore, more GWAS studies would be helpful to identify the genomic regions governing nutritional traits in wheat and also to identify the candidate genes to develop biofortified cultivars. The present study aimed to identify the genomic region(s) associated with GFeC, GZnC, and TKW in diverse bread wheat genotypes in a range of environments through the GWAS approach and the putative candidate genes associated with the SNPs.

## Materials and methods

### Plant material and field experiments

A set of 280 genetically diverse bread wheat genotypes (Supplementary Table S1) consisting of advanced breeding lines and commercial cultivars were used for GWAS analysis. The study material in GWAS panel with 280 genotypes was selected from All India Coordinated Research Project on Wheat and Barley. The GWAS panel was evaluated at five different environments: E1-University of Agricultural Sciences, research farm, Dharwad (15° 29′ 20.71″ N, 74° 59′ 3.35″ E, 750 m AMSL), E2-ICAR-Indian Agricultural Research Institute, New Delhi (28° 38′ 30.5″ N, 77° 09′ 58.2″ E, 228 m AMSL), E3-Indian Agricultural Research Institute, Jharkhand (24° 16′ 58.4″ N, 85° 21′ 16.1″ E, 651 m AMSL), E4-ICAR-Indian Institute of Wheat and Barley, Karnal (29° 41′ 8.2644'' N, 76° 59′ 25.9692″ E, 250 m AMSL), and E5-Punjab Agricultural University, Ludhiana (30^o^ 54′ N, 75^o^ 48′ E, 247 m AMSL). The crop was sown in the first fortnight of november during the 2020–2021 *Rabi* (winter) season under irrigated condition. The genotypes were planted in an augmented block design with only the checks (DBW187, MACS6222, WH1124, and WH1142) repeated in a 2 row of 2 m length with a row spacing of 20 cm.

### Phenotyping and phenotypic data analysis

Randomly selected 20–25 spikes were harvested and bulk-threshed manually in a clean cloth bag without touching any metal to avoid contamination. Around 20 g of grain sample from each genotype were used for phenotyping GFeC and GZnC through high-throughput Energy Dispersive X-ray Fluorescence (ED-XRF) machine (model X-Supreme 8000; Oxford Instruments plc, Abingdon, United Kingdom) calibrated with glass beads-based values. To record TKW, the Numigral grain counter was used to count the grain number, the reading was set at 1000 grains and the weight of the grains was recorded in grams with an electronic balance. Phenotypic data were analysed using the R package ‘augmentedRCBD’^52^. Coefficient of variation (CV), broad-sense heritability (h^2^_BS_), genotypic variance (σ^2^_G_), and environmental variance (σ^2^_E_) were calculated using the following formula:

$$\mathrm{CV }(\%)= \mathrm{SD}/\overline{\mathrm{x}}\times 100$$

where SD = Standard deviation; x̅ = Arithmetic mean.

$$LSD={t}_{0.025,{DF}_{w}}\sqrt{{MS}_{w}\left(1/{n}_{1}+1/{n}_{2}\right)}$$

where ${t}_{0.025,{DF}_{w}}$ = The t-critical value from the t-distribution table with α = 0.025 and DF_w_ is the degrees of freedom within groups from the ANOVA table. ${MS}_{w}$ = The mean squares within groups from the ANOVA table. ${n}_{1}$ and ${n}_{2}$ = The sample sizes for the first and second comparing samples

$$\mathrm{Heritability }\left({h}_{BS}^{2}\right)=\frac{{\upsigma }_{G}^{2}}{{\upsigma }_{G}^{2}+\frac{{\upsigma }_{E}^{2}}{\mathrm{nBlock}}}\times 100$$

where ${\upsigma }_{G}^{2}$ = Genetic variance was calculated as (MS_treatments_ – MS_residuals_)/ nBlock; ${\upsigma }_{E}^{2}$ = Residual variance = MS_residual_; nBlock = Number of blocks

$${\upsigma }_{G}^{2}= \frac{{MS}_{treatments}- {MS}_{residuals}}{b}$$

$${\upsigma }_{E}^{2}={MS}_{residuals}$$

where ${MS}_{treatments}$ = Treatment mean sum of square; ${MS}_{residuals}$ = Error mean sum of square; b = Number of blocks.

The CV indicates the degree of precision with which the treatments are compared and is a good index of the experimental reliability. It expresses the experimental error as percentage of the mean and if the value is high then the precision of the experiment is low and vice versa. The h^2^_BS_ is the proportion of phenotypic variation that is attributable to an overall genetic variation for the genotypes. LSD is the value at a particular level of statistical probability, when exceeded by the difference between two genotypes means, then the two genotypes are said to be distinct for at that or lesser levels of probability. The σ^2^_G_ is the genetic or inherent variation that remains unaltered by environmental changes, this kind of variation responds to the selection during breeding process. In contrast, σ^2^_E_ does not respond to selection as it is non-heritable, which is entirely due to environmental effects.

### Genotyping

Genomic DNA of the GWAS panel was extracted from the leaves of 21 days-old seedlings by Cetyl Trimethyl Ammonium Bromide (CTAB) method^53^. The panel was genotyped using Axiom Wheat Breeder’s Genotyping Array (Affymetrix, Santa Clara, CA, United States) having 35,143 genome-wide SNPs. The monomorphic, markers with minor allele frequency (MAF) of < 5%, missing data of > 20%, and heterozygote frequency > 25% were removed from the analysis. The remaining set of 14,790 high-quality SNPs was used in GWAS analysis (Supplementary Table S3).

### Population Statistics and GWAS

The pair-wise LD values (*r*^2^) between the SNPs located in each chromosome were calculated with Trait Analysis by aSSociation Evolution and Linkage (TASSEL) version 5.0^54^. The LD block size in three different subgenomes as well as in the whole genome was calculated by keeping *r*^*2*^ threshold at half LD decay (Fig. 3). The principal component analysis (PCA) was done through GAPIT^55^ to understand the structure of the population and included in the GWAS model to correct the structure. Furthermore, Kinship relationship was calculated through GAPIT^55^ and presented in Fig. 2C. Additionally, the structure of the population was evaluated through the STRUCURE program by keeping K-value from 1 to 10. For every single *K*-value, 3 independent runs were used and each run was set with 10,000 burn-in iterations followed by 10,000 Markov Chain Monte Carlo (MCMC) replications after burn-in. The STRUCTURE HARVESTER^56^ was used to detect the optimal K-value based on ad-hoc method described by Pritchard et al*.* 2010^90^ as well as Evanno’s method^57^. The suitability of the model to account for population structure was assessed using quantile–quantile (Q–Q) plots.

The phenotypic values of GFeC, GZnC, and TKW of 280 diverse genotypes along with corresponding genotyping data were used in GWAS analysis. Significant MTAs were identified using BLINK (Bayesian-information and Linkage-disequilibrium Iteratively Nested Keyway) model^58^ implemented in Genome Association and Prediction Integrated Tool (GAPIT) version 3.0^80^ in R software package. Determining the correct *P*-value threshold for statistical significance is critical to differentiate true positives from false positives. To determine the statistical significance threshold in GWAS, Bonferroni correction has been employed. To estimate Bonferroni correction, α was set to 0.05 and which is divided by total number of SNPs. The Bonferroni-corrected SNPs were considered for significant association and *R*^2^ was used to describe the percentage variation explained (PVE) by significant MTAs.

### In silico analysis

The sequence information of the significant SNPs was used to search for putative candidate genes with Basic Local Alignment Search Tool (BLAST) using default parameters in the Ensemble Plants database (http://plants.ensembl.org/index.html) of the bread wheat genome (IWGSC (RefSeq v1.0)). The genes found in the overlapping region and within the region of 10 Kb intervals flanking either side of the associated marker were considered as putative candidate genes and their molecular functions were determined. In addition, their expression patterns were investigated using the Wheat Expression database (http://www.wheat-expression.com/) and potential links to phenotypes was determined using Knetminer tool integrated with Wheat Expression database. The role of the identified putative candidate genes in the regulation of GZnC and GFeC, and TKW was also determined with the previous reports.

## Results

### Variability, heritability, and correlations

The environment-wise heritability and variance components of the GWAS panel for GFeC, GZnC, and TKW are presented in Table 1. The GFeC ranged from 26.3 mg/kg to 49.9 mg/kg, whereas, the GZnC recorded a wider distribution across the environments, as it ranged from 21.3 mg/kg to 64.1 mg/kg. Similarly, TKW ranged from 26.0 gm to 59.3 gm. The trait-wise heritability was recorded highest for TKW followed by GFeC, and GZnC, whereas, the trend for the coefficient of variation (CV) was exactly opposite with the lowest recorded for TKW followed by GFeC, and GZnC. The environment-wise heritability was ranged from 45.4% (E4) to 89.7% (E1), 33.3% (E4) to 84.9% (E5), and 89.9% (E4) to 98.8% (E1) respectively, for GFeC, GZnC, and TKW. For all the three traits, E4 has been recorded as the lowest heritability, which was corroborated with the highest recorded CV for E4. The genotypic variance (σ^2^_G_) and environmental variance (σ^2^_E_) are presented in Table 1.

**Table 1:** Descriptive statistics, variance and heritability estimates of grain quality traits in GWAS panel evaluated at five environments during 2020–2021.

Trait	Env.	Mean ± SD	Range	CV (%)	LSD	*h*_bs_^2^	σ_G_^2^	σ_E_^2^
GFeC (mg/kg)	E1	39.5 ± 3.62	31.9–49.0	3.0	3.3	89.7	12.1	1.4
	E2	37.8 ± 2.85	30.6–47.5	2.9	3.1	83.6	6.2	1.2
	E3	40.7 ± 3.1	32.2–49.9	4.1	4.7	71.3	6.7	2.7
	E4	35.3 ± 4.02	26.3–47.9	7.2	7.2	45.4	5.4	6.4
	E5	40.2 ± 3.49	32.4–49.8	3.1	3.5	87.7	10.9	1.5
GZnC (mg/kg)	E1	39.6 ± 7.29	22.6–62.5	9.9	11.1	67.8	32.4	15.4
	E2	43.8 ± 5.26	32.8–57.8	5.4	6.7	77.7	19.5	5.6
	E3	35.2 ± 5.91	21.3–52.9	13.1	13.0	34.8	11.3	21.2
	E4	36.9 ± 4.02	28.6–47.6	8.7	9.0	33.3	5.0	10.1
	E5	45.9 ± 8.44	24.7–64.1	6.9	9.1	84.9	57.9	10.3
TKW (gm)	E1	45.6 ± 5.17	29.6–58.2	1.3	1.6	98.8	26.9	0.3
	E2	41.3 ± 3.65	30.3–51.0	2.4	2.8	92.7	12.4	1.0
	E3	46.1 ± 4.17	34.9–59.3	2.2	2.8	94.2	16.4	1.0
	E4	44.6 ± 4.35	29.9–57.9	3.1	3.9	89.9	17.1	1.9
	E5	40.5 ± 4.11	26.0–49.0	1.6	1.9	97.4	16.3	0.4

GFeC: grain iron concentration; GZnC: grian zinc concentration; TKW: thousand kernel weight; E1-Dharwad; E2-IARI,New Delhi; E3-IARI, Jharkhand; E4-Karnal; E5-Ludhiana; Env.: Environment; SD: standard deviation; CV: coefficient of variation; *h*^*2*^bs: broad sense heritability; σ^2^_G_: genotypic variance; σ^2^_E_: environmental variance.

**Figure 1:**
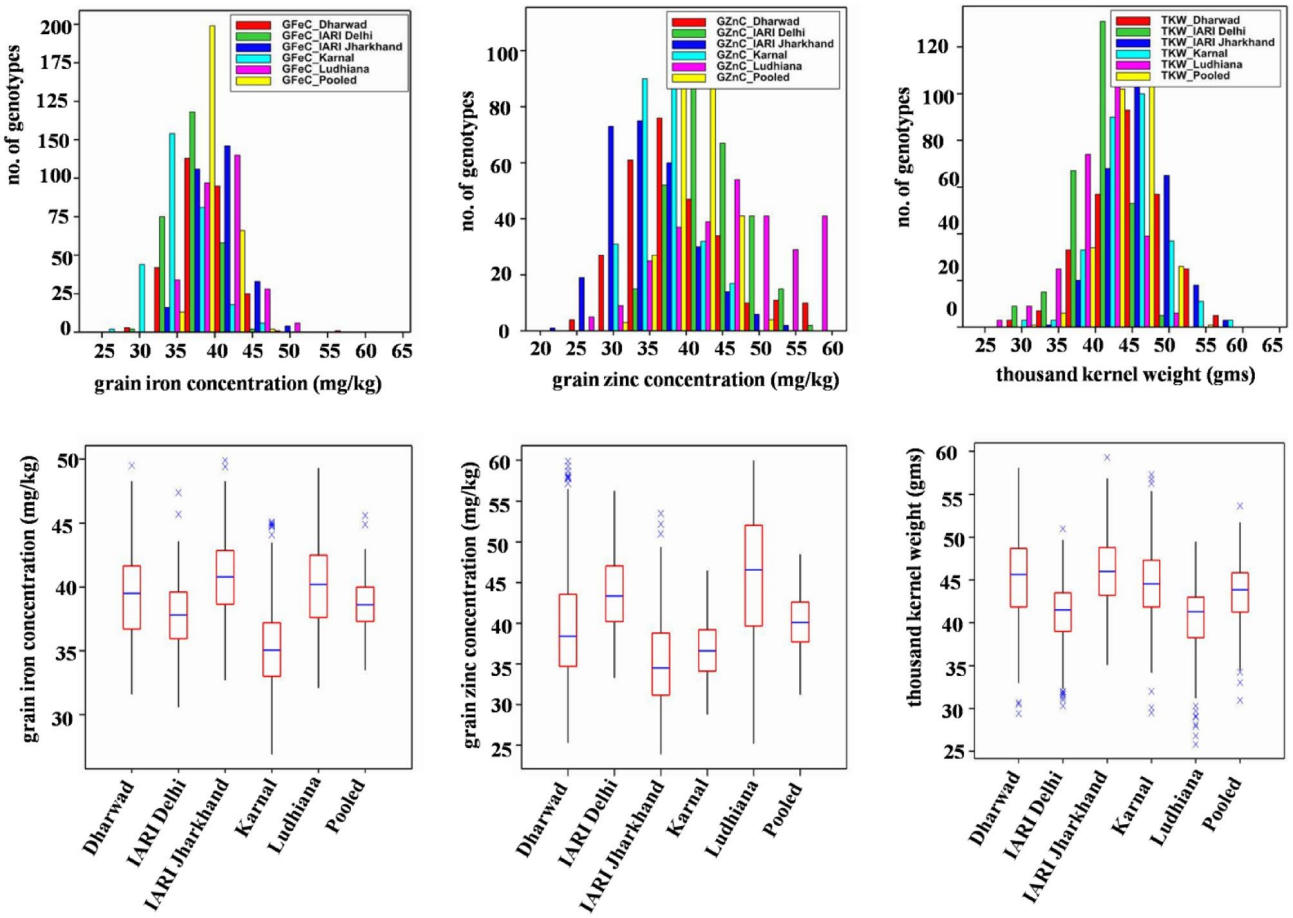
Frequency distribution and boxplots of grain quality traits in GWAS panel evaluated at Dharwad, IARI Delhi, IARI Jharkhand, Karnal, and Ludhiana during 2020–2021.

The trait and environment-wise mean values are illustrated graphically through boxplots and presented in Fig. 1. The location means of GFeC were recorded as similar and highest for E3 and E5 followed by E1, E2, and E4, whereas, E5 was recorded highest pooled mean followed by E2, E1, E4, and E3 for GZnC. The E3 and E1 recorded a similar and highest mean for TKW followed by E4, E2, and E5. The frequency distribution of grain quality traits in the GWAS panel evaluated at E1–E5 during 2020–2021 is presented in Fig. 1. The genotypes in the GWAS panel showed continuous frequency distributions for all the studied traits. Partial correlation coefficient (*r*^*2*^) of GFeC, and GZnC by keeping TKW as a controlling factor was determined. Highly significant and positive correlation was observed between GFeC and GZnC in E1 (0.296**), E2 (0.276**), E3 (0.202**), E4 (0.520**), and E5 (0.35**) and also in pooled data (0.358**).

### SNP markers statistics

The quality processing of 35,143 SNPs from 35 K array resulted in a set of 14,790 cured genome-wide SNPs. These high-quality set of SNPs were further used for GWAS analysis. The chromosome and genome-wise marker distribution are presented in Table 2. The highest number of SNPs were mapped on the B genome (5649) followed by the D genome (4590), and the A genome (4551).

**Table 2:** Sub-genome and chromosome-wise distribution of SNP markers in the GWAS panel.

Genome	Chromosome-wise SNP distribution							Total
	1	2	3	4	5	6	7	
A	751	756	587	493	699	515	750	4551
B	1077	992	726	465	863	766	760	5649
D	986	951	648	264	657	459	625	4590

### Population structure and linkage disequilibrium

**Figure 2:**
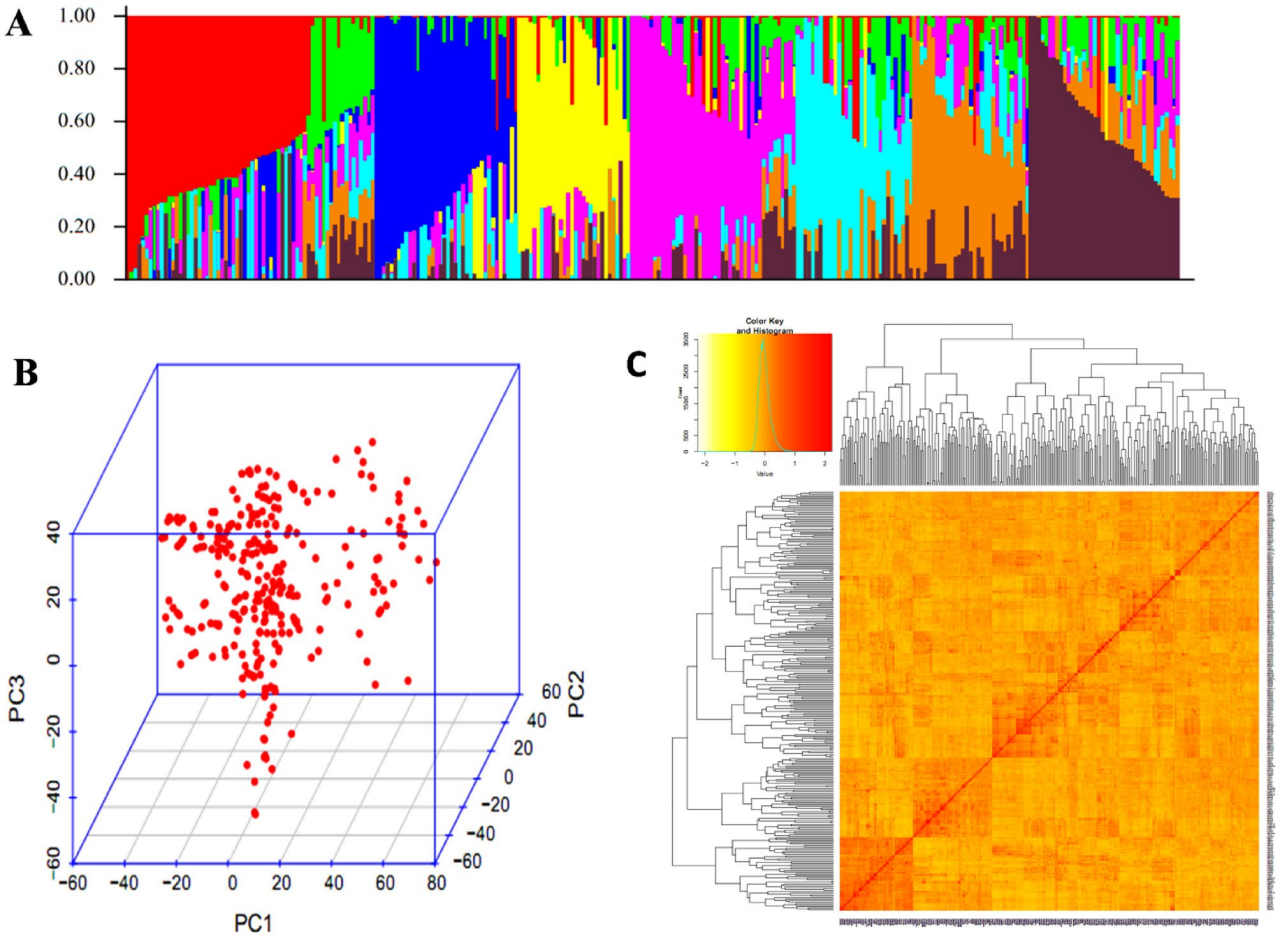
Population groupings in GWAS panel from different models. (**A**) Population structure based on STRUCTURE  (**B**) Three-dimensional plot of the first three principal components, and (**C**) heat map of pair-wise kinship matrix.

**Figure 3:**
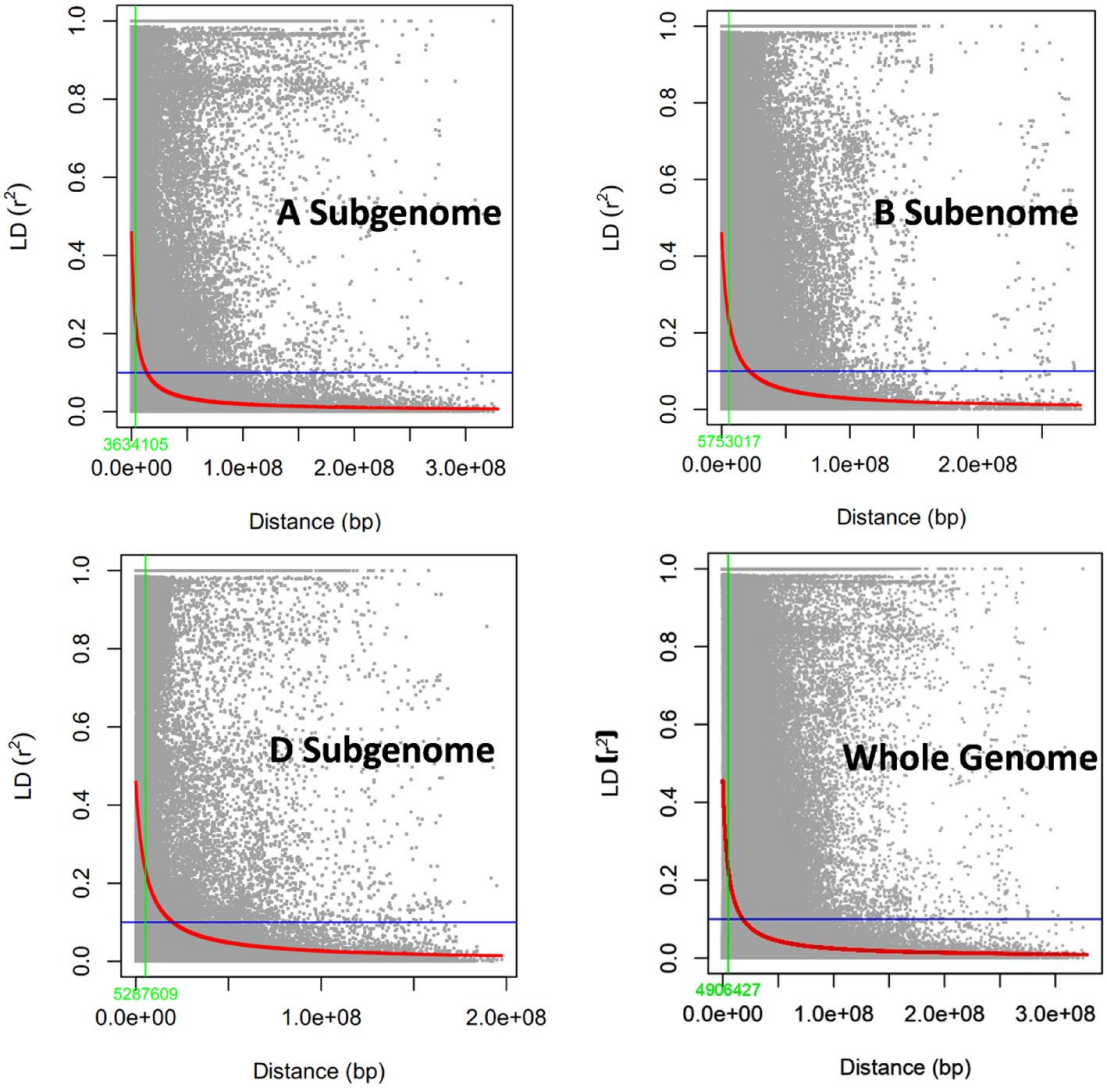
Subgenome and whole genome-wide linkage disequilibrium (LD) decay in GWAS panel of 280 diverse bread wheat genotypes.

The PCA plot (Fig. 2B) indicated that there were no clear distinct sub-populations in the GWAS panel; however, STRUCTURE grouped the GWAS panel into eight sub-populations (Fig. 2A). The LD was estimated by calculating the squared correlation coefficient (r^2^) for all the SNPs and plotted against the genetic distance (bp). The LD decay for the whole genome was 4.9 cM and it was found that the decay was rapid in the A subgenome (3.6 cM) followed by the B subgenome (5.7 cM) and the D subgenome (5.2 cM) (Fig. 3).

### Genome-wide association studies

**Figure 4:**
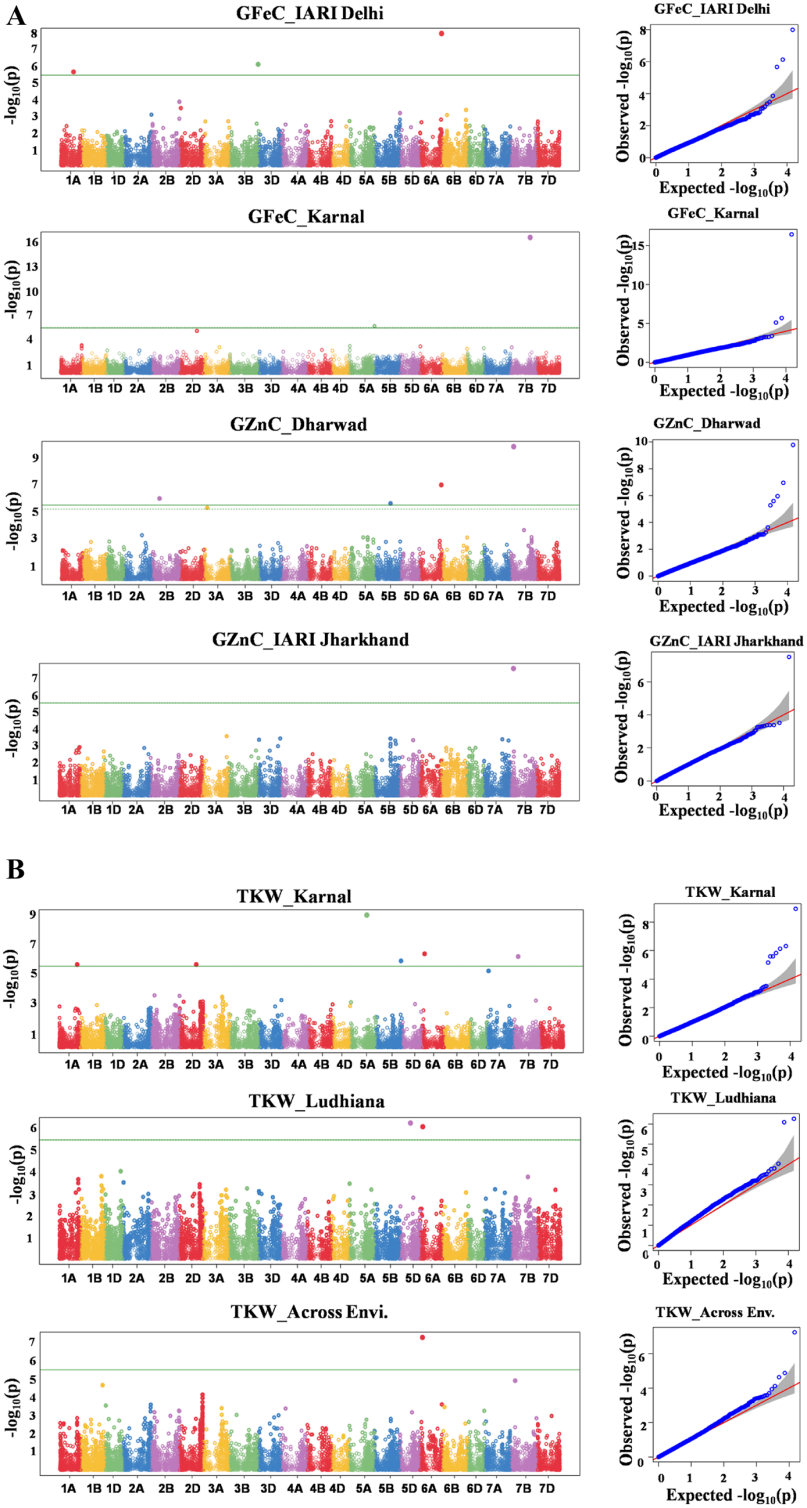
(**A**) Manhattan and respective-QQ plots for grain iron and zinc concentration in GWAS panel phenotyped at Dharwad, IARI Delhi, IARI Jharkhand, Karnal, and Ludhiana during 2020–2021. (**B**) Manhattan and respective-QQ plots for thousand kernel weight in GWAS panel phenotyped at Dharwad, IARI Delhi, IARI Jharkhand, Karnal, and Ludhiana during 2020–2021.

A total of 17 Bonferroni-corrected MTAs were identified for GFeC, GZnC, and TKW. The details of the identified MTAs are presented in Table 3 and illustrated in Manhattan plots in Fig. 4A,B. The Q-Q plots depicting the observed associations of SNPs and GFeC, GZnC, and TKW compared to the expected associations after accounting for population structure are presented in Fig. 4A,B.

**Table 3:** MTAs for grain quality traits and TKW identified in the GWAS panel from five environments.

Trait	Env	SNPs	Chromosome	Position (bp)	*P *value	PVE (%)
GFeC	E2	*AX-94423274*	6A	609111057	1.02E−08	15.6
	E2	*AX-94490975*	3B	795800318	0.000000747	12.7
	E2	*AX-95195514*	1A	354950241	0.00000214	13
	E4	*AX-94699865*	7B	558319306	3.69E−17	24.1
	E4	*AX-95140213*	5A	706021202	0.00000203	23.1
GZnC	E1	*AX-95118780*	7B	91660994	1.68E−10	10.9
	E1	*AX-95113687*	6A	595578858	1.11E−07	10.1
	E1	*AX-94390652*	2B	201463130	1.10E−06	5.7
	E1	*AX-94524014*	5B	440179428	2.55E−06	8.8
	E3	*AX-95203413*	7B	94271868	3.06E−08	10
TKW	E4	*AX-94764034*	5A	444849916	1.19E−09	16.1
	E4	*AX-95025823*	6A	68975107	0.00000049	16.1
	E4	*AX-94452219*	7B	131745573	0.000000747	14.9
	E4	*AX-94820753*	5B	689950369	0.00000147	13.7
	E4	*AX-94569403*	2D	461303027	0.00000256	17.4
	E4	*AX-95235178*	1A	499807792	0.00000257	16.7
	E5	*AX-95117294*	5D	290389058	0.000000555	10.7
	E5	*AX-95025823*	6A	68975107	0.000000821	11.7
	Across Env	*AX-95025823*	6A	68975107	5.83E−08	16.9

Env.: Environment; GFeC: grain iron concentration; GZnC: grian zinc concentration; TKW: thousand kernel weight; E1: Dharwad; E2: IARI Delhi; E3: IARI Jharkhand; E4: Karnal; E5: Ludhiana; SNPs: single nucleotide polymorphisms; PVE: phenotypic variation explained.

### MTAs for GFeC and GZnC

A total of five significant MTAs were identified for GFeC in E2 and E4 environments on chromosomes 6A, 3B, 1A, 7B, and 5A explaining the phenotypic variation ranged from 12.7% to 24.1%. Two major SNPs (*AX-9469986* and *AX-95140213*) on 7B and 5A chromosomes located at 706.0 Mb and 558.3 Mb explained the highest phenotypic variation of 24.1% and 23.1%, respectively in E4 environment. One SNP each on chromosome 6A (*AX-94423274*), 3B (*AX-94490975*), and 1A (*AX-95195514*) were mapped at 609.1 Mb, 795.8 Mb, and 354.9 Mb, respectively with the phenotypic variation of 15.6%, 12.7%, and 13.0% in E2 environment.

A total of 5 MTAs were identified for GZnC on chromosome 7B, 6A, 2B, 5B, and 7B explaining the phenotypic variation ranged from 5.7% to 10.9%. The B subgenome contributed more MTAs (4) followed by A subgenome (1), whereas, D subgenome didn’t contribute for GZnC in the present study. Two major SNPs (*AX-95118780* and *AX-95140213*) on 7B chromosome located at 91.6 Mb and 94.2 Mb explained the highest phenotypic variation of 10.9% and 10.0%, respectively in E1 and E3 environments. Another major SNP (*AX-95113687*) on the A subgenome (6A chromosome) mapped at 595.5 Mb, explained 10.1% phenotypic variation in E1. The remaining two SNPs (*AX-94390652* and *AX-94524014*) on 2B and 5B chromosomes mapped at 201.4 Mb and 440 Mb explained 5.7% and 8.8% phenotypic variation, respectively in E1.

### MTAs for TKW

A total of seven MTAs were identified covering all the subgenomes. Major phenotypic variation was observed from those MTAs which were ranging from 10.7% to 17.4%. The three subgenomes mapped more or less the same number of MTAs (A subgenome-3, B and D subgenomes-2 each). Six MTAs (*AX-94764034, AX-95025823, AX-94452219, AX-94820753, AX-94569403,* and *AX-95235178*) were identified in E4 on chromosome 5A, 6A, 7B, 5B, 2D, and 1A at 444.8 Mb, 68.9 Mb, 131.7 Mb, 689.9 Mb, 461.3 Mb, and 499.8 Mb, respectively with a corresponding phenotypic variation of 16.1%, 16.1%, 14.9%, 13.7%, 17.4%, and 16.7%. A total of 2 MTAs (*AX-95117294* and *AX-95025823*) were mapped in E5 located at 290.3 Mb and 68.9 Mb, which explained 10.7% and 11.7% phenotypic variation on 5D and 6A, respectively.

For pooled TKW data, one MTA (*AX-95025823*) was mapped on 6A and located at 68.9 Mb, which explained 16.9% phenotypic variation. One stable MTA i.e. *AX-95025823 *was identified in both E4 and E5 environments along with pooled data, which is located at 68.9 Mb on 6A chromosome.

### Identification of putative genes associated with MTAs

The significant SNPs associated with GFeC, GZnC, and TKW were used to identify the putative candidate genes using the annotated wheat reference sequence (RefSeq V1.0) and are presented in Table 4 and Supplementary Table 2. *AX-94490975* associated with GFeC found to encode *Multi antimicrobial extrusion protein* (TraesCS3B02G562500). Similarly, another SNP i.e. *AX-94699865* associated with GFeC encodes an important *F-box domain *(TraesCS7B02G312400). Two important SNPs i.e. *AX-94524014* (TraesCS5B02G257700) and *AX-95203413* (TraesCS7B02G083600) associated with GZnC were found to encode *Late embryogenesis abundant protein, LEA-18* and *RNA recognition motif domain.* Similarly, *AX-95235178* encoding *Leucine-rich repeat domain superfamily (*TraesCS1A02G309000) and *AX-95117294* encoding *C3H4 TYPE ZINC FINGER PROTEIN* (TraesCS5D02G188300) identified for TKW.

**Table 4:** Putative candidate genes identified for GFeC, GZnC, and TKW along with their molecular functions.

Trait	SNP ID	Chromosome	TransID	Position (bp)	Putative candidate genes	Molecular function
GFeC	*AX-94490975*	3B	TraesCS3B02G562500	795800216–795810270	Multi antimicrobial extrusion protein	Iron translocation in bread wheat^59^ Efficient translocation of iron from roots to shoots in rice^60^ Iron homoeostasis in Arabidopsis^61,81,82^ Fe homeostasis and Zn tolerance in Arabidopsis^62^ Fe influx into aerial parts of the plant and/or for the distribution of intracellular Fe^63,64^ Aluminum tolerance and iron translocation in *Arabidopsis thaliana*^65^ Iron transportation in rice^83^ Efficient translocation of Fe under limited Fe conditions in rice^60^ Efficient translocation of iron in Arabidopsis^84^ Overexpression of *MtMATE69* affected Fe and Zn accumulation in *M. truncatula* and play role in Fe nutrition^85^ Iron efficiency in soybean^86^
	*AX-94699865*	7B	TraesCS7B02G312400	558318164–558320180	F-box domain	F-box domain RAE1 regulates STOP1 in Arabidopsis. STOP1-ALMT1 pathway promote iron accumulation into the apoplast of root tip regions under Pi-deficient conditions^66,67^
GZnC	AX-94524014	5B	TraesCS5B02G257700	440178901–440179528	Late embryogenesis abundant protein, LEA-18	Iron transportation in the phloem of castor (*Ricinus communis* L.)^68^ Zinc ion binding in cotton^69^
TKW	AX-95235178	1A	TraesCS1A02G309000	499807479–499809182	Leucine-rich repeat domain superfamily	Regulate grain size in rice^71^ Role in total kernel number and kernel size in maize^87^ Controls endosperm development and thereby seed size^88^
	AX-95117294	5D	TraesCS5D02G188300	290386100–290391792	C3H4 TYPE ZINC FINGER PROTEIN	Important agronomic traits in maize yield^89^

## Discussion

Understanding the genetic basis of complex traits such as GFeC, GZnC, and TKW through GWAS with a diverse panel of genotypes can significantly improve QTL mapping resolution compared to bi-parental populations-based QTL mapping. Using the genome-wide SNPs and multi-environment data, several significant SNPs were identified in this study.

The expression of GFeC, GZnC, and TKW is significantly affected by the environment and genotype-environment interactions (GEI). Among all traits, GZnC was the most environment-sensitive trait, whereas, TKW was relatively the most stable with minimum environmental influence. The greater magnitude of the environment and GEI have also been reported in previous studies for the expression of GFeC and GZnC^10,11^, and TKW^12,13^. The magnitude of environmental interaction decides the identification of environment-specific QTL(s) as well as QTL(s) that can express stably across environments.

The highest heritability was recorded for TKW followed by GFeC, and GZnC, whereas, the trend for the coefficient of variation (CV) was exactly opposite with the lowest CV recorded for TKW and the highest CV for GZnC. The highest and lowest heritability for TKW and GZnC respectively is also concurred with earlier studies^46,72^. The associations were highly significant positive in all the environments betweenGFeC and GZnC. Significant and positive correlations found in this study have also been reported in earlier studies^25,26^. The significant positive correlations between GZnC and GFeC indicated the possibility to map the genomic regions controlling multiple traits. Such co-mapped SNPs will be much useful in marker-assisted selection for simultaneous improvement of correlated traits.

The STRUCTURE model explained 8 sub-groups in the populations. The genotypes in GWAS panel consists of advanced breeding lines suitable for various agro-climatic and production conditions. The first subgroup consists of genotypes mostly selected from international breeding material and suited for North West and North East Plains Zone in India. Similarly, the second group consists of international selections for restricted irrigated or rainfed production conditions. The third subgroup consists of genotypes dominated by 1B.1R translocation with genes for wider adaptation. Subpopulation 4 is mainly dominated by GW322, PASTOR, and OPATA parentage, whereas, 5th subpopulation largely consists of Indian wheat varieties/germplasm in their parentage. High frequency of SOKOLL, KIRITATI, PBW65, and MILAN was present in the 6th subpopulation parentage. Genotypes in 7th subpopulation are dominated by old salinity/alkalinity tolerant varieties. Whereas, 8th subpopulation contains mainly indigenous germplasm, old landraces, and breeding lines. The PC1, PC2 and PC3 of PCA analysis were used as covariates in the GWAS analysis to identify the MTAs. The LD may vary in different populations due to population size, genetic drift, admixtures, selection, mutation, non-random mating, pollination behavior, and recombination frequency^73,74^. The LD blocks are usually larger in self-pollinated crops such as wheat and hence decay slowly^75^, whereas, in outcrossing crop species like maize^76^, the LD decays rapidly. The presence of high LD across the genome would reduce the QTL mapping resolution and vice versa^77^. In such cases, a better QTL resolution will be achieved by using genome-wide SNPs. The decay of LD was found comparable in the B and D subgenomes (~ 5 cM) compared to the A subgenome, which had a shorter decay distance of around ~ 3 cM. A similar pattern of LD decay was also observed in other GWAS studies in wheat^49,78,79,91^.

A total of 17 Bonferroni-corrected MTAs were identified for GFeC (5), GZnC (5), and TKW (7). The identified genome-wise MTAs are much higher for B subgenome (8) and A subgenome (7) compared to the D subgenome (2). A similar trend on MTAs identified in the D subgenome for GFeC and GZnC^41^ and yield-contributing traits^49,50^.

The identified MTAs (5) for GFeC on chromosomes 6A, 3B, 1A, 7B, and 5A in this study were novel, as the earlier reported MTAs on the same chromosomes namely 3B, 7B, and 5A^43,44^, 1A, 3B, and 5A^23,26^, 1A^40^ and 6A, 3B^91^ were identified at different positions. A total of five novel MTAs was identified for GZnC on chromosome 7B, 6A, 2B, 5B, and 7B. MTAs in the same chromosomes were also identified in different GWAS panels in previous experiments on 6A^47^, 2B^26,41,43,47^, 5B^23,43,47^, and 7B^23,41,91^. Zhou et al.^47^ identified an MTA on 5B chromosome located in a interval of 407.0 Mb – 412.1 Mb, which was similar to that of *AX-94524014* located on 5B chromosome and mapped at 440.1 Mb explained 8.8% phenotypic variation.

A total of seven MTAs in different environments were identified covering the three subgenomes and all were major MTAs as they explained more than 10.0% phenotypic variation. The TKW was relatively the most stable trait compared to the rest of the other two traits, as TKW recorded the highest heritability and lowest coefficient of variation which reflected in detecting the highest number of MTAs as well. All the identified MTAs were mapped on 5A, 6A, 7B, 5B, 2D, 7A, and 5D located at 444.8 Mb, 68.9 Mb, 131.7 Mb, 689.9 Mb, and 461.3 Mb, 499.8 Mb, and 290.3 Mb respectively. Previous reports were also identified MTAs on 6A and 7B^29,48,50^, 5B^26,49^, 5A^26^, 1D^29^, 1A^48^ and 2D, 5D, 7A and 7B^91^.

The various putative candidate genes underlying MTAs with high phenotypic variation for GZnC, GFeC, and TKW were identified through BLAST search (Table 4 and Supplementary Table 2). The MTAs identified in various chromosomes were located in gene coding regions related to transcription factors, transporters, transmembrane protein and kinase-like superfamilies. For example, *Multi antimicrobial extrusion protein* (TraesCS3B02G562500) has a role in the translocation of iron during iron deficiency stress in bread wheat^59^ and multi antimicrobial extrusion protein (MATE) family proteins were observed under iron excess in rice. Few protein members of MATE family were known to be involved in efficient iron translocation from roots to shoots in rice^60^. Also, MATE transporter mediates iron homoeostasis under osmotic stress in Arabidopsis^61^. Subfamily III of the MATE gene members plays an important role in plant aluminum tolerance and iron translocation in Arabidopsis^65^. FRD3 MATE transporter locus reveals cross-talk between Fe homeostasis and Zn tolerance in Arabidopsis by loading Zn into xylem^62^. MATE is also a candidate for the mechanism of Fe influx into aerial parts of the plant and the distribution of intracellular Fe^63,64^.

Three up-regulated genes i.e. *Os01g0684900, Os10g0345100,* and *Os06g0495500* of citrate transporters family (MATE family protein) were observed under excess iron conditions and involved in iron transportation in rice^83^. Similarly, a MATE gene (*OsFRDL1*), the closest homolog of barley *HvAACT1* (aluminum-activated citrate transporter 1) is involved in the efficient translocation of Fe under limited Fe conditions^60^. FRD3 is a member of the multidrug and toxin efflux (MATE) family, which is involved in the efficient translocation of iron in Arabidopsis^84^. FRD3 is mainly expressed in root vascular tissues and is necessary to solubilize Fe and Zn in the extracellular space. Similarly, overexpression of *MtMATE69* affected Fe and Zn accumulation in *Medicago truncatula* hairy roots, further suggesting a function for *MtMATE69* in Fe nutrition^85^. Also, two MATE proteins namely, *GmFRD3a* and *GmFRD3b* play a significant role in iron efficiency in soybean^86^. Cloning and characterization of an Arabidopsis gene i.e. *FRD3*, a member of the multidrug and toxin efflux family is involved in iron homeostasis^81^. The *FRD3*, which is an efflux transporter of the efficient Fe chelator citrate is involved in Fe homeostasis maintenance throughout plant growth and development. Additionally to its well-known root expression, *FRD3* is also strongly expressed in seeds and flowers^82^.

One SNP i.e. *AX-94699865* associated with GFeC encodes an important *F-box domain* (TraesCS7B02G312400) regulates STOP1 in Arabidopsis. STOP1-ALMT1 pathway promotes iron accumulation into the apoplast of root tip regions under Pi-deficient conditions^66,67^. Another SNP i.e. *AX-94524014* (TraesCS5B02G257700) associated with GZnC was found to encode *LEA protein, where LEA-18 was* involved in the transportation of iron in the phloem of castor^68^. The binding of LEA proteins to different molecules like Zn ion, DNA and ATP binding, were the major activities for the action of upland LEA proteins^69^.

The SNP *i.e. AX-95235178* encoding *Leucine-rich repeat domain superfamily (*TraesCS1A02G309000) was associated with TKW. A total of 32 barley orthologs were identified as potential candidate genes that determine barley grain size or weight. The barley ortholog of the rice *OsBDG1* gene is mapped on 3H chromosome at 666.35 Mb (*HORVU3Hr1G104350*), which encodes the leucine-rich repeat receptor-like protein kinase family^70^. The rice *OsBDG1* gene encoding a small protein with short leucine-rich-repeats possessing cell elongation activity, has previously been proven to positively regulate grain size in rice^71^. Therefore, *HORVU3Hr1G104350*could be a reliable candidate gene affecting grain size as the function of the *OsBDG1* gene.

Another grain weight controlling gene i.e. *FASCIATED EAR2* (*FEA2*) encodes the maize ortholog of *CLAVATA2* (*CLV2*), encoding a leucine-rich repeat receptor-like protein that regulates meristem size by transmitting signals from *CLAVATA3* (*CLV3*) peptide ligand to the *WUSCHEL* (*WUS*) homeodomain transcription factor. The *FEA2* has a role in total kernel number and kernel size in maize^87^. Similarly, IKU pathway represents one of the well-studied genetic networks involves four major genes including *HAIKU2 (IKU2)*, which encodes a leucine-rich repeat kinase, mutational analyses of these genes in Arabidopsis revealed their physiological significance in controlling endosperm development and thereby seed size through regulating endosperm proliferation and cellularization^88^, and loss of function mutations in IKU pathway genes cause a decrease in seed size. Another SNP (*AX-95117294*) encoding C3H4 type zinc finger protein (TraesCS5D02G188300) was associated with the expression of TKW. Functional prediction of maize C2H2—zinc finger gene revealed its involvement mainly in the formation of important agronomic traits in maize yield^89^.

The study with 280 diverse set of bread wheat GWAS panel has shown that GFeC, GZnC, and TKW were quantitatively inherited traits. The strong positive correlation between the GFeC and GZnC suggested the possibility of improving both the traits simultaneously. A total of 17 MTAs including 5 for GFeC, 5 for GZnC, and 7 for TKW were identified from the GWAS approach. The environment-specific and pooled-data MTAs identified in the present investigation represented novel genomic regions associated with trait expression. Several putative candidate genes encoding important molecular functions such as iron translocation, iron and zinc homeostasis, and grain size modifications were associated with the identified MTAs. Further validation and functional characterization of the candidate genes to elucidate the role of these genes in wheat is envisaged. The identified SNPs could be useful in marker-assisted selection programs to develop biofortified varieties to reduce micronutrient malnutrition.

### Declaration

The set of 280 genotypes used in the present experiment were selected from All India Coordinated Research Project on Wheat and Barley and the imported genotypes have been obtained through the nodal agency for germplasm exchange i.e. National Bureau of Plant Genetic Resources, New Delhi following the prescribed guidelines. Also, the authors have all the required permissions and rights to collect and use the genotypes for research purpose. The experimental research and field experiments in the present study are duly approved by the institute research council of ICAR-Indian Institute of Wheat and Barley Research, Karnal.

## Supplementary Information

Supplementary Information 1.

Supplementary Information 2.

Supplementary Information 3.

## Data Availability

All the phenotypic and genotypic data used in the study is given as Supplementary Table 3 and also deposited in DRYAD (an international open-access repository of research data). The international open-access data repository link is as follows: https://datadryad.org/stash/share/FaLrCt8Hd4sKOqzWyGSNYQ0Q_tGtdSKIE3a9rBYAVTM.
