# Supplementary material for: Genetic dissection of grain iron and zinc, and thousand kernel weight in wheat (Triticum aestivum L.) using genome-wide association study
Source: Sci Rep. 2022 Jul 20;12:12444. doi: 10.1038/s41598-022-15992-z (PMC9300641; doi:10.1038/s41598-022-15992-z)
Supplement: Supplementary file 2 — Supplementary Information 2. [file 41598_2022_15992_MOESM2_ESM.docx]

**Supplementary Table 2.** Putative candidate genes identified for GFeC, GZnC, and TKW

| **Trait** | **SNP ID** | **Chr.** | **TransID** | **Position (bp)** | **Putative candidate genes (overlapping/nearby)** |
| --- | --- | --- | --- | --- | --- |
| GFeC | AX-94423274 | 6A | TraesCS6A02G398500 | 609,109,331-609,112,709 | F7O18.3 PROTEIN |
|  | *AX-94490975* | 3B | TraesCS3B02G562500 | 795,800,216-795,810,270 | Multi antimicrobial extrusion protein |
|  | *AX-95195514* | 1A | [TraesCS1A02G196800](http://plants.ensembl.org/Triticum_aestivum/Gene/Summary?db=core;g=TraesCS1A02G196800;r=6A:609111018-609111095;t=TraesCS1A02G196800.1;tl=GzSF0InvM0V3WGMj-21419396-2311847689) | [354,950,050-354,955,890](http://plants.ensembl.org/Triticum_aestivum/Location/View?db=core;g=TraesCS1A02G196800;r=1A:354950050-354955890;t=TraesCS1A02G196800.1;tl=GzSF0InvM0V3WGMj-21419396-2311847689) | Calcium permeable stress-gated cation channel |
|  | *AX-94699865* | 7B | [TraesCS7B02G312400](http://plants.ensembl.org/Triticum_aestivum/Gene/Summary?db=core;g=TraesCS7B02G312400;r=6A:609111018-609111095;t=TraesCS7B02G312400.1;tl=GzSF0InvM0V3WGMj-21419397-2311868609) | [558,318,164-558,320,180](http://plants.ensembl.org/Triticum_aestivum/Location/View?db=core;g=TraesCS7B02G312400;r=7B:558318164-558320180;t=TraesCS7B02G312400.1;tl=GzSF0InvM0V3WGMj-21419397-2311868609) | F-box domain |
|  | *AX-95140213* | 5A | TraesCS5A02G553700 | 706,020,414-706,022,749 | EXPRESSED PROTEIN |
| GZnC | AX-95118780 | 7B | TraesCS7B02G081200 | [91,660,098-91,663,931](http://plants.ensembl.org/Triticum_aestivum/Location/View?db=core;g=TraesCS7B02G081200;r=7B:91660098-91663931;t=TraesCS7B02G081200.1;tl=GzSF0InvM0V3WGMj-21419399-2311848185) | Transport inhibitor response 1 domain |
|  | AX-94390652 | 2B | [TraesCS2B02G216100](http://plants.ensembl.org/Triticum_aestivum/Gene/Summary?db=core;g=TraesCS2B02G216100;r=6A:609111018-609111095;t=TraesCS2B02G216100.1;tl=GzSF0InvM0V3WGMj-21419401-2311847721) | [201,462,877-201,473,257](http://plants.ensembl.org/Triticum_aestivum/Location/View?db=core;g=TraesCS2B02G216100;r=2B:201462877-201473257;t=TraesCS2B02G216100.1;tl=GzSF0InvM0V3WGMj-21419401-2311847721) | Armadillo-type fold |
|  | AX-94524014 | 5B | [TraesCS5B02G257700](http://plants.ensembl.org/Triticum_aestivum/Gene/Summary?db=core;g=TraesCS5B02G257700;r=5B:440178901-440179528;t=TraesCS5B02G257700.1) | [440,178,901-440,179,528](http://plants.ensembl.org/Triticum_aestivum/Location/View?db=core;g=TraesCS5B02G257700;r=5B:440178901-440179528;t=TraesCS5B02G257700.1) | Late embryogenesis abundant protein, LEA-18 |
|  | AX-95203413 | 7B | TraesCS7B02G083600 | 94,267,847-94,271,780 | RNA recognition motif domain |
| TKW | AX-94764034 | 5A | [TraesCS5A02G228900](http://plants.ensembl.org/Triticum_aestivum/Gene/Summary?db=core;g=TraesCS5A02G228900;r=5B:440178901-440179528;t=TraesCS5A02G228900.1;tl=GzSF0InvM0V3WGMj-21419404-2311868620) | [444,849,640-444,851,597](http://plants.ensembl.org/Triticum_aestivum/Location/View?db=core;g=TraesCS5A02G228900;r=5A:444849640-444851597;t=TraesCS5A02G228900.1;tl=GzSF0InvM0V3WGMj-21419404-2311868620) | Peptidase C13, legumain |
|  | AX-95025823 | 6A | TraesCS6A02G101000.1 | 68,974,484-68,974,793 | Protein coading |
|  | AX-94452219 | 7B | [TraesCS7B02G113500](http://plants.ensembl.org/Triticum_aestivum/Gene/Summary?db=core;g=TraesCS7B02G113500;r=5B:440178901-440179528;t=TraesCS7B02G113500.2;tl=GzSF0InvM0V3WGMj-21419406-2311847859) | [131,739,073-131,745,621](http://plants.ensembl.org/Triticum_aestivum/Location/View?db=core;g=TraesCS7B02G113500;r=7B:131739073-131745621;t=TraesCS7B02G113500.2;tl=GzSF0InvM0V3WGMj-21419406-2311847859) | Ubiquitin-like domain |
|  | AX-94820753 | 5B | [TraesCS5B02G532600](http://plants.ensembl.org/Triticum_aestivum/Gene/Summary?db=core;g=TraesCS5B02G532600;r=5B:440178901-440179528;t=TraesCS5B02G532600.2;tl=GzSF0InvM0V3WGMj-21419407-2311868660) | [689,944,087-689,951,241](http://plants.ensembl.org/Triticum_aestivum/Location/View?db=core;g=TraesCS5B02G532600;r=5B:689944087-689951241;t=TraesCS5B02G532600.2;tl=GzSF0InvM0V3WGMj-21419407-2311868660) | CASTOR/POLLUX/SYM8 ion channel, conserved domain |
|  | AX-94569403 | 2D | [TraesCS2D02G359400](http://plants.ensembl.org/Triticum_aestivum/Gene/Summary?db=core;g=TraesCS2D02G359400;r=5B:440178901-440179528;t=TraesCS2D02G359400.1;tl=GzSF0InvM0V3WGMj-21419408-2311868667) | [461,301,196-461,307,149](http://plants.ensembl.org/Triticum_aestivum/Location/View?db=core;g=TraesCS2D02G359400;r=2D:461301196-461307149;t=TraesCS2D02G359400.1;tl=GzSF0InvM0V3WGMj-21419408-2311868667) | Domain of unknown function DUF3741 |
|  | AX-95235178 | 1A | TraesCS1A02G309000 | 499,807,479-499,809,182 | Leucine-rich repeat domain superfamily |
|  | AX-95117294 | 5D | TraesCS5D02G188300 | 290,386,100-290,391,792 | C3H4 TYPE ZINC FINGER PROTEIN |
